# EGFR amplification and outcome in a randomised phase III trial of chemotherapy alone or chemotherapy plus panitumumab for advanced gastro-oesophageal cancers

**DOI:** 10.1136/gutjnl-2020-322658

**Published:** 2020-11-16

**Authors:** Elizabeth C Smyth, Georgios Vlachogiannis, Somaieh Hedayat, Alice Harbery, Sanna Hulkki-Wilson, Massimiliano Salati, Kyriakos Kouvelakis, Javier Fernandez-Mateos, George D Cresswell, Elisa Fontana, Therese Seidlitz, Clare Peckitt, Jens C Hahne, Andrea Lampis, Ruwaida Begum, David Watkins, Sheela Rao, Naureen Starling, Tom Waddell, Alicia Okines, Tom Crosby, Was Mansoor, Jonathan Wadsley, Gary Middleton, Matteo Fassan, Andrew Wotherspoon, Chiara Braconi, Ian Chau, Igor Vivanco, Andrea Sottoriva, Daniel E Stange, David Cunningham, Nicola Valeri

**Affiliations:** 1 Department of Oncology, Cambridge University Hospitals NHS Foundation Trust, Cambridge, UK; 2 Department of Medicine, Royal Marsden Hospital NHS Trust, London, UK; 3 Molecular Pathology, The Institute of Cancer Research, Sutton, UK; 4 Centre for Evolution and Cancer, The Institute of Cancer Research, Sutton, UK; 5 Cancer Therapeutics, The Institute of Cancer Research, Sutton, UK; 6 Clinical Research & Development, Royal Marsden Hospital NHS Trust, London, UK; 7 Department of Visceral, Thoracic and Vascular Surgery, University Hospital Carl Gustav Carus, Dresden, Germany; 8 Department of Medical Oncology, Christie Hospital, Manchester, UK; 9 Department of Clinical Oncology, Velindre Cancer Centre, Cardiff, UK; 10 Cancer Clinical Trials Centre, Weston Park Cancer Centre, Sheffield, UK; 11 Institute of Immunology and Immunotherapy, University of Birmingham, Birmingham, UK; 12 Department of Medicine (DIMED), University of Padua, Padova, Italy; 13 Histopathology, Royal Marsden Hospital NHS Trust, London, UK; 14 Institute of Cancer Sciences, University of Glasgow, Glasgow, UK; 15 German Cancer Consortium (DKTK), Partner Site Dresden, Heidelberg, Germany; 16 National Center for Tumor Diseases, Partner Site Dresden, Heidelberg, Germany

**Keywords:** gastric adenocarcinoma, gastrointestinal cancer, molecular oncology, oesophageal cancer

## Abstract

**Objective:**

Epidermal growth factor receptor (EGFR) inhibition may be effective in biomarker-selected populations of advanced gastro-oesophageal adenocarcinoma (aGEA) patients. Here, we tested the association between outcome and *EGFR* copy number (CN) in pretreatment tissue and plasma cell-free DNA (cfDNA) of patients enrolled in a randomised first-line phase III clinical trial of chemotherapy or chemotherapy plus the anti-EGFR monoclonal antibody panitumumab in aGEA (NCT00824785).

**Design:**

*EGFR* CN by either fluorescence in situ hybridisation (n=114) or digital-droplet PCR in tissues (n=250) and plasma cfDNAs (n=354) was available for 474 (86%) patients in the intention-to-treat (ITT) population. Tissue and plasma low-pass whole-genome sequencing was used to screen for coamplifications in receptor tyrosine kinases. Interaction between chemotherapy and EGFR inhibitors was modelled in patient-derived organoids (PDOs) from aGEA patients.

**Results:**

*EGFR* amplification in cfDNA correlated with poor survival in the ITT population and similar trends were observed when the analysis was conducted in tissue and plasma by treatment arm. EGFR inhibition in combination with chemotherapy did not correlate with improved survival, even in patients with significant *EGFR* CN gains. Addition of anti-EGFR inhibitors to the chemotherapy agent epirubicin in PDOs, resulted in a paradoxical increase in viability and accelerated progression through the cell cycle, associated with p21 and cyclin B1 downregulation and cyclin E1 upregulation, selectively in organoids from *EGFR*-amplified aGEA.

**Conclusion:**

*EGFR* CN can be accurately measured in tissue and liquid biopsies and may be used for the selection of aGEA patients. EGFR inhibitors may antagonise the antitumour effect of anthracyclines with important implications for the design of future combinatorial trials.

**Significance of this study**

**What is already known on this subject?**

- Clinical trials combining epidermal growth factor receptor (EGFR) inhibitors and chemotherapy for advanced gastro-oesophageal adenocarcinomas (GEAs) have failed in unselected patient populations.
- Anecdotal data suggest that *EGFR* amplification might predict benefit from EGFR inhibitors in advanced GEA.
- Heterogeneous expression of biomarkers is an intrinsic characteristic of GEA and analysis of liquid biopsies as a tool to overcome intrapatient heterogeneity is warranted.

**What are the new findings?**

- We tested *EGFR* copy number in tissue and liquid biopsies from advanced GEA patients enrolled in a prospective randomised phase III trial of chemotherapy alone or chemotherapy plus the anti-EGFR monoclonal antibody panitumumab.
- *EGFR* status could be reliably detected in tissue and liquid biopsies and concordance between the two was observed in 95% of cases.
- *EGFR* amplification in tissue and circulating cell-free DNA appeared as a negative prognostic marker in the intention-to-treat population and in individual treatment arms.
- *EGFR*-amplified cases treated with EGFR inhibitors in combination with chemotherapy had a particularly poor prognosis. Using patient-derived organoids, we showed an antagonistic effect between anti-EGFR agents and the chemotherapy drug epirubicin specifically in *EGFR*-amplified organoids.

**Significance of this study**

**How might it impact on clinical practice in the foreseeable future?**

- Our study demonstrates the robustness and feasibility of biomarker testing in liquid biopsies of advanced gastro-oesophageal adenocarcinoma (GEA) patients. Liquid biopsies could be used in prospective trials for advanced GEA patients as a screening tool.
- Epidermal growth factor receptor inhibitors should not be combined with chemotherapy regimens containing anthracyclines with significant implications in GEA and other tumour types.
- Patient-derived preclinical models are of paramount importance in defining mechanisms of action of drug combinations.

## Introduction

Amplification of receptor tyrosine kinases is a hallmark of gastric and oesophageal adenocarcinomas, with epidermal growth factor receptor (EGFR) amplification occurring in 6%–10% of gastro-oesophageal adenocarcinomas (GEAs).^1–3^ Although there is level 1 evidence supporting treatment of GEA and breast tumours which harbour amplifications of human epidermal growth factor receptor 2 (HER2) with anti-HER2 therapy, the effect of targeting the EGFR axis in *EGFR*-amplified tumours is less well established.^4–6^ Two large randomised trials, REAL3 and EXPAND, evaluated the efficacy of addition of anti-EGFR monoclonal antibodies (mAb) to cytotoxic chemotherapy in patients with unselected, treatment naïve advanced GEA.^7 8^ Neither trial demonstrated an improvement in survival for anti-EGFR treated patients in a non-biomarker selected patient population. However, emerging translational analyses from unselected clinical trials and new early phase trials in biomarker-defined subgroups suggest that a population of *EGFR*-amplified GEA exist who might benefit from anti-EGFR therapy.^2 9^ If future clinical trials seek to evaluate the efficacy of anti-EGFR therapy in biomarker-enriched populations, understanding the interaction between *EGFR* amplification and chemotherapy outcome is essential for rational trial design. Additionally, as heterogeneous expression of biomarkers is a fundamental characteristic of gastro-oesophageal cancers, analysis of liquid biopsies as a tool to overcome intrapatient heterogeneity is warranted.^10 11^

In this study, we tested the association between *EGFR* amplification and clinical outcome in patients treated with epirubicin, oxaliplatin and capecitabine plus or minus panitumumab (EOX±P) in the REAL3 (randomised trial of EOX with or without panitumumab in Advanced or Locally Advanced Oesophagogastric Cancer 3) Trial (NCT00824785). *EGFR* status was tested by digital-droplet PCR (ddPCR) and fluorescent in situ hybridisation (FISH) in tumour samples and by ddPCR in plasma samples, and was correlated with progression free (PFS) and overall survival (OS) in the trial population. Patient-derived organoids (PDOs) from *EGFR*-amplified and non-amplified metastatic GEA patients^12 13^ were used for reverse translation in order to study the interaction between chemotherapy and EGFR inhibitors.

## Methods

### REAL3 Trial Population

Details of the REAL3 trial have been previously described.^8^ REAL3 eligible patients had a diagnosis of locally advanced or metastatic oesophagogastric cancer and were treated with EOX (epirubicin, oxaliplatin and capecitabine) plus or minus the fully human monoclonal IgG_2_ anti-EGFR antibody panitumumab. Patients treated with EOX plus panitumumab had inferior OS compared with patients treated with EOX (HR 1.37, 95% CI 1.07 to 1.76; p=0.013). From the REAL3 patient population (n=553), pretreatment tumour biopsies (tissue blocks) with high tumour content (>30%) were selected by a pathologist. All patients included in this analysis had given informed consent for translational research. DNA was extracted from formalin fixed paraffin embedded (FFPE) pretreatment tumour biopsies using QIAamp DNA FFPE (Qiagen, Manchester, UK). Cell-free DNA (cfDNA) was extracted from two ml of patient plasma using the QIAamp DNA Blood Mini Kit (Qiagen, Manchester, UK). cfDNA was eluted in 50 µL of elution buffer and quantified using the Qubit dsDNA HS Assay Kit (Thermo Fisher Scientific, Loughborough, UK).

### Statistical methods

The objectives of the study were to test: (1) the sensitivity and specificity of *EGFR* testing by FISH vs ddPCR; (2) the correlation between *EGFR* status in solid (tissue) versus liquid (pretreatment cfDNA) biopsies; (3) the association between *EGFR* amplification, PFS and OS in the intention to treat population (ITT) and in each arm of the study separately and (4) descriptive analysis of PFS and OS in *EGFR* amplified cases based on treatment arm.

We defined *EGFR* amplification as *EGFR*/*CNTNAP2* ratio of ≥2 using ddPCR. Exploratory analyses also evaluated the effect of *EGFR* amplification when a higher cut-off was chosen for amplification (ddPCR ratio in tissue or plasma of ≥5.0). PFS and OS were estimated using the Kaplan-Meier method. Groups were compared using the log-rank test and Cox regression was used to generate HRs and 95% CIs. Response rates were compared between groups using logistic regression, which generated ORs and 95% CIs. In multivariate analysis, forward stepwise Cox regression was used to calculate corrected HRs and 95% CIs. Statistical analyses were performed using Stata V.13 (Timberlake Consultants, Richmond on Thames, UK).

Online supplemental methods can be found online.

10.1136/gutjnl-2020-322658.supp1 Supplementary data

## Results

### Study population and EGFR copy number analysis

**Figure 1:**
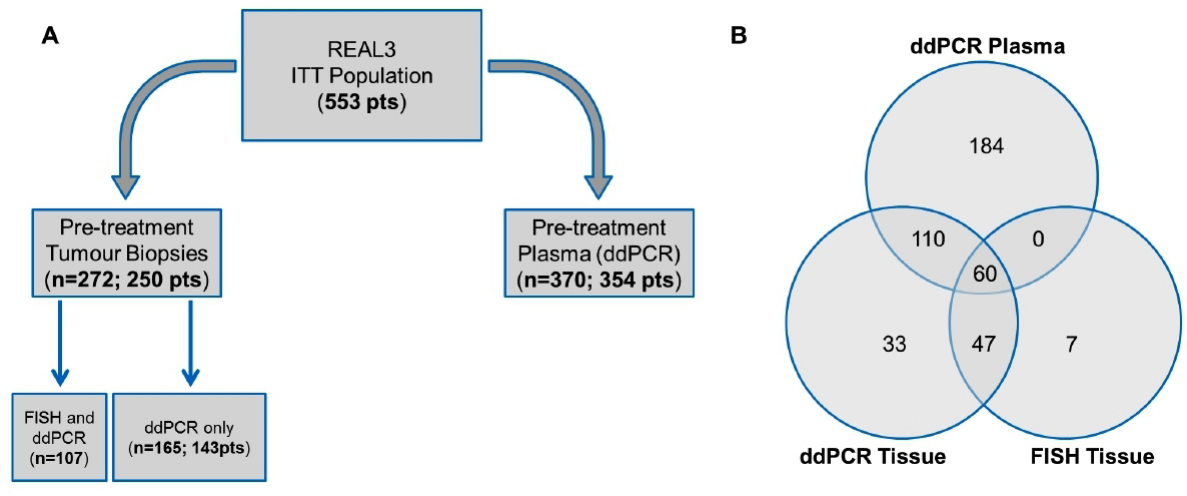
Tissue and liquid biopsy analysis in the REAL3 trial. (A) Diagram shows the number of patients for whom FFPE tissues and plasma cfDNA were available for *EGFR* testing. (B) Venn diagram shows the number of patients tested for *EGFR* amplification based on source of material (FFPE tissues vs cfDNA) and method used (ddPCR vs FISH). cfDNA, cell free DNA; ddPCR, digital-droplet PCR; EGFR, epidermal growth factor receptor; FFPE, formalin-fixed paraffin embedded; FISH, fluorescent in situ hybridisation; ITT, intention to treat.

Of the 553 REAL3 participants, 272 tissue samples from 250 patients and 370 pretreatment cfDNA samples from 354 patients were available for *EGFR* copy number variation (CNV) analysis either by FISH or ddPCR (figure 1A). In all patients with multiple tissue or cfDNA samples available, a 100% concordance in *EGFR* CNV was observed across samples.

*EGFR* CNV FISH data on chemonaïve pretreatment tissue (resections or biopsies) were available in 114 out of 200 patients enrolled in the phase II portion of the REAL3 trial; reasons for no availability of results included: (1) lack of patient consent for analysis (n=10); (2) insufficient tumour tissue left following previous analyses (n=36); (3) technical failure (n=18); (4) other reasons for example, block returned to treating hospital (n=22). Of the 114 samples successfully tested, nine patients (7.9%) were found positive for *EGFR* amplification by FISH.

Using ddPCR, *EGFR* CNV score on pretreatment tissue was ≥2.0 in 17/250 (6.8%), and ≥5.0 in 12/250 (4.8%) patients. DdPCR *EGFR* CNV score on pretreatment cfDNA was ≥2.0 in 22/354 (6.2%) and ≥5.0 in 10/354 (2.8%) patients. Overall, cfDNA concentration appeared higher in metastatic compared with locally advanced cancers (p=0.08), while no significant differences in cfDNA concentration were observed based on gender, localisation of the primary cancer (oesophagus, junction or stomach), or based on presence of lung or peritoneal metastases. A small but significant difference in cfDNA concentration was, however, observed between patients with or without liver metastases (p=0.007) (online supplemental table 1).

10.1136/gutjnl-2020-322658.supp2 Supplementary data

### Comparative EGFR CN analysis in solid and liquid biopsies

Comparison between *EGFR* CNV determined by FISH and ddPCR on tissue was available in 107 cases, while comparison between *EGFR* CNV determined by ddPCR both on tissue and cfDNA was possible in 170 cases. Finally, comparison among *EGFR* CNV determined by ddPCR on tissue and cfDNA, as well as by FISH on tissue, was available in 60 cases. Overall, *EGFR* CNV status using either FISH, or ddPCR on tissue or cfDNA, was available in 474 patients (85.7% of the ITT population) (figure 1B).

In situ hybridisation is considered the gold standard for the determination of *HER2* CNV status in breast and gastro-oesophageal cancer^14^; we, therefore, used a similar approach for *EGFR* CNV status, and considered FISH as the reference. Concordance between *EGFR* CNV assessed by ddPCR and FISH on tumour tissue was 98%. Compared with FISH, ddPCR showed sensitivity of 1 (95% CI 0.61 to 1.00), specificity of 0.97 (95% CI 0.92 to 0.99), positive predictive value of 0.81 (95% CI 0.47 to 0.96), and negative predictive value of 0.95 (95% CI 0.95 to 1.00) (online supplemental table 2). Next, we compared *EGFR* CNV between matched pretreatment tissue and cfDNA samples, assessed by ddPCR. Overall, concordance was 94.7% (online supplemental table 3); six cases that scored as *EGFR*-amplified on tissue (3 of them with more than 12 *EGFR* copies) showed no amplification in cfDNA, while three cases showed *EGFR* amplification in cfDNA but not on the matched tissue. No significant differences in cfDNA concentration were observed in patients with or without *EGFR* amplification, suggesting that variable input of cfDNA is unlikely responsible for these discrepancies (online supplemental figure 1). Finally, when *EGFR* status was tested in the 60 cases for which data by FISH and ddPCR on tissue, and by ddPCR on cfDNA were available, concordance between tissue and blood was 95%: 4 cases scored as *EGFR*-amplified and 53 cases as *EGFR* non-amplified by all the three methods, while in 3 cases an *EGFR* amplification was detected in tissue (by both FISH and ddPCR) but not in cfDNA (online supplemental table 4).

### EGFR CN in tissue and prognosis in the REAL3 trial

Given the high concordance between *EGFR* status assessed by FISH and ddPCR on tissue samples, we performed a survival analysis on the 250 patients for whom ddPCR *EGFR* CNV data on tissue were available. Patient characteristics based on *EGFR* status in each treatment arm are shown in table 1. Patients whose tumours harboured *EGFR-*amplification (ddPCR CNV score ≥2) had a median PFS of 4.57 months (95% CI 2.5 to 10.56 months), compared with 6.41 months (95% CI 5.99 to 7.34 months) for non-amplified cases (HR 1.30 (95% CI 0.78 to 2.16); p=0.32); a similar trend was observed when a higher threshold (ddPCR CNV score ≥5) was used for identifying *EGFR*-amplification (HR 1.70 (95% CI 0.92 to 3.12) p:0.09) (online supplemental table 5). Similar observations were drawn when the PFS analysis was conducted by treatment arm using both cut-offs (online supplemental table 6). In multivariate analysis adjusting for sex, age, performance status, disease status (locally advanced vs metastatic), and histological subtype, *EGFR* amplification retained a negative prognostic value regardless of treatment arm (EOX arm HR 1.3 (95% CI 0.5 to 2.9); EOX-P=1.3 (95% CI 0.7 to 2.5)).

**Table 1:** Patient characteristics by treatment arm and *EGFR* status determined on tumour tissue (n=250)

	EOX arm		EOX-P arm	
	EGFR non-amplified (<2)	EGFR amplified (≥2)	EGFR non-amplified (<2)	EGFR amplified (≥2)
Median age (IQR)	62.6 (54.2–68.4)	63.8 (54.3–68.6)	63.2 (56.6–69.7)	63.1 (59.0–65.4)
Males (%)	97 (80.8)	7 (100)	89 (78.8)	10 (100)
Females (%)	23 (19.2)	–	24 (21.2)	–
PS 0 (%)	44 (36.7)	4 (57.1)	42 (37.2)	5 (50)
PS 1 (%)	69 (57.5)	2 (28.6)	65 (57.5)	5 (50)
PS 2 (%)	7 (5.8)	1 (14.3)	6 (5.3)	–
Locally advanced (%)	8 (6.7)	–	10 (8.9)	1 (10)
Metastatic (%)	112 (93.3)	7 (100)	103 (91.1)	9 (90)

EGFR, epidermal growth factor receptor; EOX, epirubicin, oxaliplatin, capecitabine; P, panitumumab; PS, performance status.

Patients with *EGFR*-amplified tumours (ddPCR CNV score ≥2) also had a lower median OS of 9.74 months (95% CI 3.68 to 11.35 months), compared with 11.18 months for patients with *EGFR* non-amplified tumours (95% CI 8.78 to 13.16 months) (HR 1.28 (95% CI 0.77 to 2.13); p=0.35). A similar trend was observed when a higher cut-off (ddPCR CNV score ≥5) was applied in order to identify *EGFR* amplification (HR 1.5 (95% CI 0.82 to 2.79); p=0.47) (online supplemental table 7). Analogous results were observed when the OS analysis was conducted by treatment arm for both cut-offs (online supplemental table 8).

Given the small number of *EGFR*-amplified cases in our trial cohort limited the statistical power of the analysis, we sought to validate our findings in an independent cohort of GEA. We screened the cBioportal for Cancer Genomics^15 16^ for localised and metastatic GEA cases for whom *EGFR* amplification status was available. Our search returned data on 2122 samples from 2054 patients (online supplemental figure 2A) across 11 non-overlapping studies. In line with our findings, *EGFR*-amplification was observed in approximately 6.5% of patients and was consistently associated with inferior OS, disease-specific survival, disease-free survival and PFS (online supplemental figure 2B-E) in the validation cohorts. Taken together these data strengthen the notion that EGFR-amplification may be a prognostic biomarker in GEA.

### EGFR CN in circulating cfDNA and prognosis in the REAL3 trial

**Figure 2:**
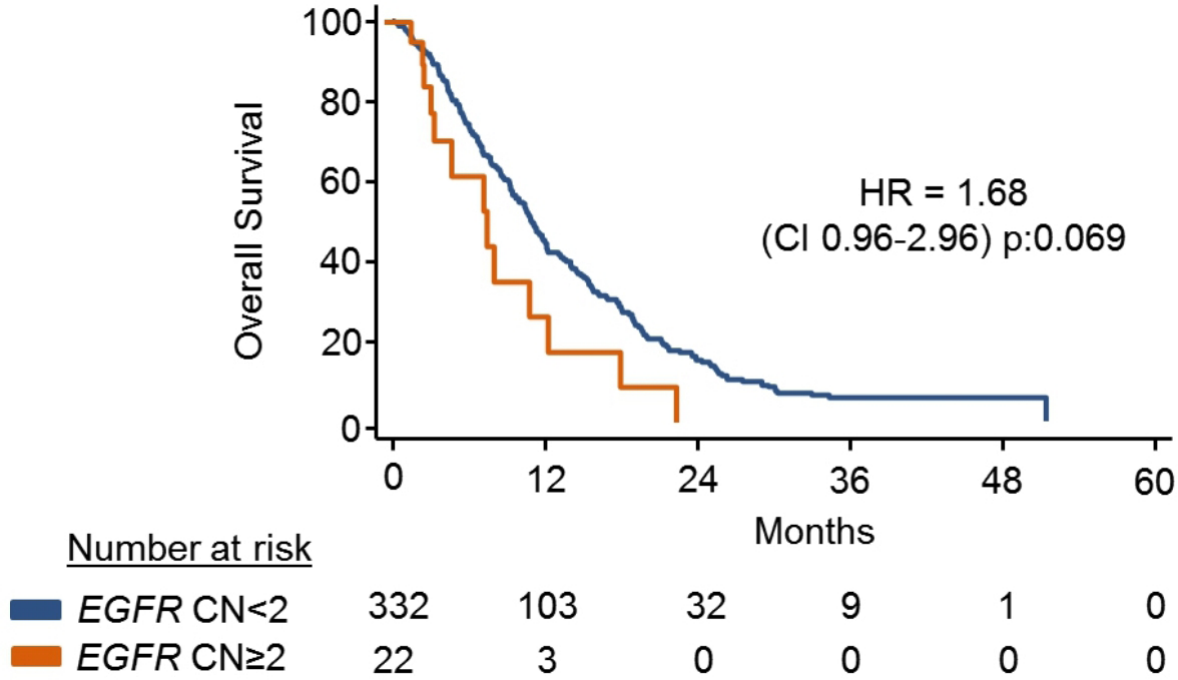
Overall survival (OS) based on *EGFR*-amplification in the REAL3 trial. *EGFR* CN was determined using ddPCR on pretreatment cfDNA. Kaplan-Meier curves show the OS of patients based on the presence/absence of *EGFR* CN. cfDNA=cell free DNA; CN, copy number; ddPCR, digital-droplet PCR; EGFR, epidermal growth factor receptor;

*EGFR* CNV was determined by ddPCR in cfDNA for 354 patients (table 2). In keeping with the results obtained from tissue, patients with *EGFR*-amplified disease had worse PFS (online supplemental table 9) and OS (figure 2; online supplemental table 10) compared with non-amplified cases. Patients with *EGFR*-amplified tumours exhibited a similar trend for worse PFS (online supplemental table 11) and OS (online supplemental table 12) also when survival analysis was conducted by treatment arm.

**Table 2:** Patients characteristics by treatment arm and *EGFR* status determined on cfDNA (n=354)

	EOX arm		EOX-P arm	
	EGFR non-amplified (<2)	EGFR amplified (≥2)	EGFR non-amplified (<2)	EGFR amplified (≥2)
Median age (IQR)	62.8 (56.1–68.3)	60.8 (49.8–65.3)	63.4 (55.8–69.9)	62.7 (50.2–67.1)
Males (%)	139 (85.3)	3 (75)	138 (81.7)	16 (88.9)
Females (%)	24 (14.7)	1 (25)	31 (18.3)	2 (11.1)
PS 0 (%)	70 (42.9)	2 (50)	77 (45.6)	10 (55.5)
PS 1 (%)	82 (50.3)	2 (50)	85 (50.30)	7 (38.9)
PS 2 (%)	11 (6.8)	–	7 (4.1)	1 (5.6)
Locally advanced (%)	20 (12.3)	–	26 (15.4)	2 (11.1)
Metastatic (%)	143 (87.7)	4 (100)	143 (84.6)	16 (88.9)

cfDNA, cell-free DNA; EGFR, epidermal growth factor receptor; EOX, epirubicin, oxaliplatin, capecitabine; P, panitumumab; PS, performance status.

### Combined analysis of the effect of EGFR CN and treatment on the outcome of patients in the REAL3 trial

**Figure 3:**
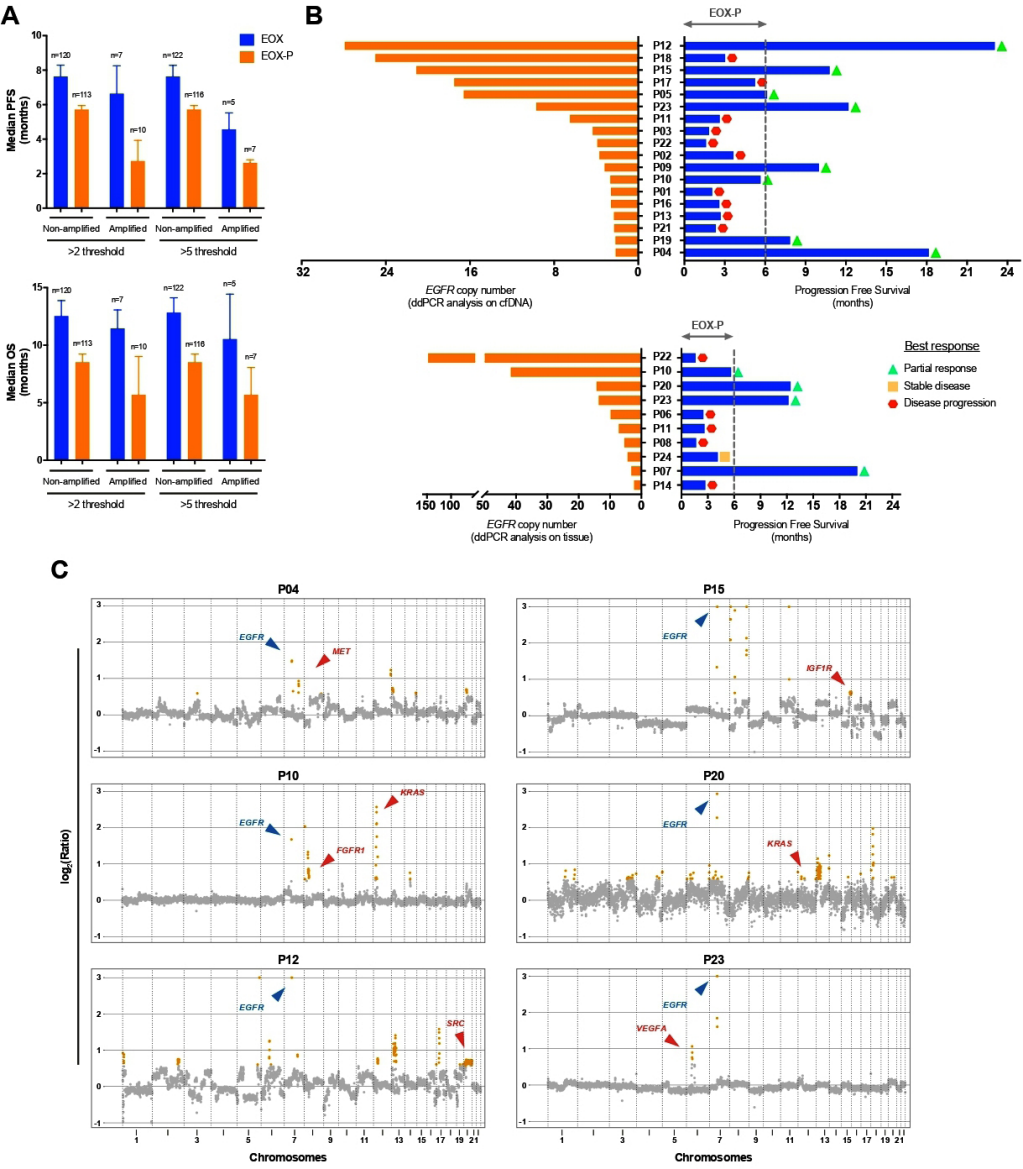
Clinical outcome by treatment arm in *EGFR*-amplified cases enrolled in the REAL3 trial. (A) Bars show OS and PFS (median±SE) in *EGFR*-amplified patients treated with chemotherapy alone (EOX) or chemotherapy plus panitumumab (EOX-P). Similar trends are observed when a cut-off of two or five *EGFR* copies is used. (B) Blue bars indicate PFS, orange bars indicate *EGFR* copies determined by ddPCR in plasma cfDNA (graph on the top) or tissue (bottom). (C) Plots show copy number changes in *EGFR* (blue) and other receptor tyrosine kinase genes (red) in patients with PFS greater than 6 months on treatment with EOX-P. Values outside of the y-axis limits are plotted at the limit. cfDNA, cell-free DNA; CN, copy number; ddPCR, digital-droplet PCR; EGFR, epidermal growth factor receptor; EOX, epirubicin+oxaliplatin+capecitabine; OS, overall survival; P, panitumumab; PFS, progression-free survival.

*EGFR* CN gains extrapolated by tissue or cfDNA analyses were overall associated with worse outcome in the REAL3 ITT population, consistent with the concept that *EGFR* amplification is an oncogenic driver in GEA. Interestingly, however, when conducted by treatment arm, our survival analyses revealed that the anti-EGFR mAb panitumumab did not mitigate this negative prognostic effect in patients with *EGFR*-amplification. In keeping with the detrimental effect of panitumumab observed in the ITT population,^8^ the combination of EGFR inhibition (EGFRi) and EOX chemotherapy was consistently associated with inferior outcome in both *EGFR* non-amplified (online supplemental table 13) and *EGFR*-amplified cases (online supplemental table 14). Despite the observation that adding panitumumab to EOX had a negative prognostic role in both groups, it was unexpected to notice that the negative association between EGFRi and EOX appeared more pronounced in patients whose tumour harboured an *EGFR* amplification (figure 3A; online supplemental table 14). Indeed, when the analysis was conducted among all the *EGFR*-amplified cases (based on plasma and cfDNA), the overall response rate was 78% in EOX vs 50% in EOX-P treated patients with a 6 months PFS of 71.4% (95% CI 44.7% to 100%) vs 39.8% (95% CI 24.1% to 65.9%) in EOX compared with EOX-P, respectively (online supplemental figure 3A, B).

Although the small number of patients in these subanalyses prevents drawing firm conclusions, it was interesting to notice that no benefit from *EGFR*i was observed even in cases with expected oncogene addiction due to significant levels of *EGFR* amplification (figure 3B). Given tyrosine kinase receptor coamplifications have been linked to benefit and resistance to EGFRi,^17^ we also tested cfDNA from *EGFR*-amplified patients treated with EOX-P who had a PFS greater than 6 months for potential drivers of response or resistance. Tissue (n=3) and circulating cfDNA (n=7) were available for 9 out of 10 patients, for one patient both tissue and circulating cfDNA were available (PT23). Low coverage whole-genome sequencing (online supplemental table 15) confirmed the presence of *EGFR* amplifications in all the cases with good correlation in CN between ddPCR and WGS (r^2^:0.82; p:0.003) for both tissue and plasma cfDNA. 70% of patients had coamplifications of receptor tyrosine kinase or other cancer-promoting genes, including *MET*, *KRAS*, *FGFR1*, *IGFR1*, *SRC* and VEGFA^18–20^, potentially associated with resistance to EGFR-inhibitors (figure 3C). Interestingly, the *VEGFA* amplification was not detected in the tissue but only in plasma for PT23 (online supplemental figure 4). Taken together, these data suggest that some of the partial responses and favourable outcomes observed in *EGFR*-amplified cases treated with EOX-P were probably driven by the EOX chemotherapy backbone alone rather than by *EGFR*i.

Inferior outcome in GEA patients treated with panitumumab in combination with an epirubicin-containing chemotherapy regimen compared with chemotherapy alone, has also been reported by the AIO/CAO STO-0801 trial.^21^ Interestingly, a similar detriment was not observed in the EXPAND trial that investigated the addition of the anti-EGFR mAb cetuximab in a comparable chemotherapy backbone that excluded the anthracycline component.^7^ Indeed, multiple lines of evidence suggest that anti-EGFR agents may be effective when combined with fluoropyrimidines and platinum compounds for the treatment of metastatic GEA cancers,^2 9^ but evidence advocating their combination with anthracyclines are lacking and, if anything, point towards an antagonistic effect between anthracyclines and EGFRi.^22^ Based on these data and prompted by the unexpected effect of the combination between panitumumab and chemotherapy in *EGFR*-amplified cases in the REAL3 trial (figure 3A), we investigated a potential antagonistic effect between epirubicin and EGFRi in GEA patients.

### PDOs model antagonisms between anthracyclines and EGFRi

The lack of commercially available *EGFR*-amplified GEA cancer cells lines has so far hindered the pre-clinical testing of EGFRi in gastric cancer. Hereafter, using *EGFR*-amplified and non-amplified PDOs established from metastatic gastric adenocarcinomas^12 13^ we provide experimental evidence of the antagonistic interaction between epirubicin and EGFRi in mGEA.

**Figure 4:**
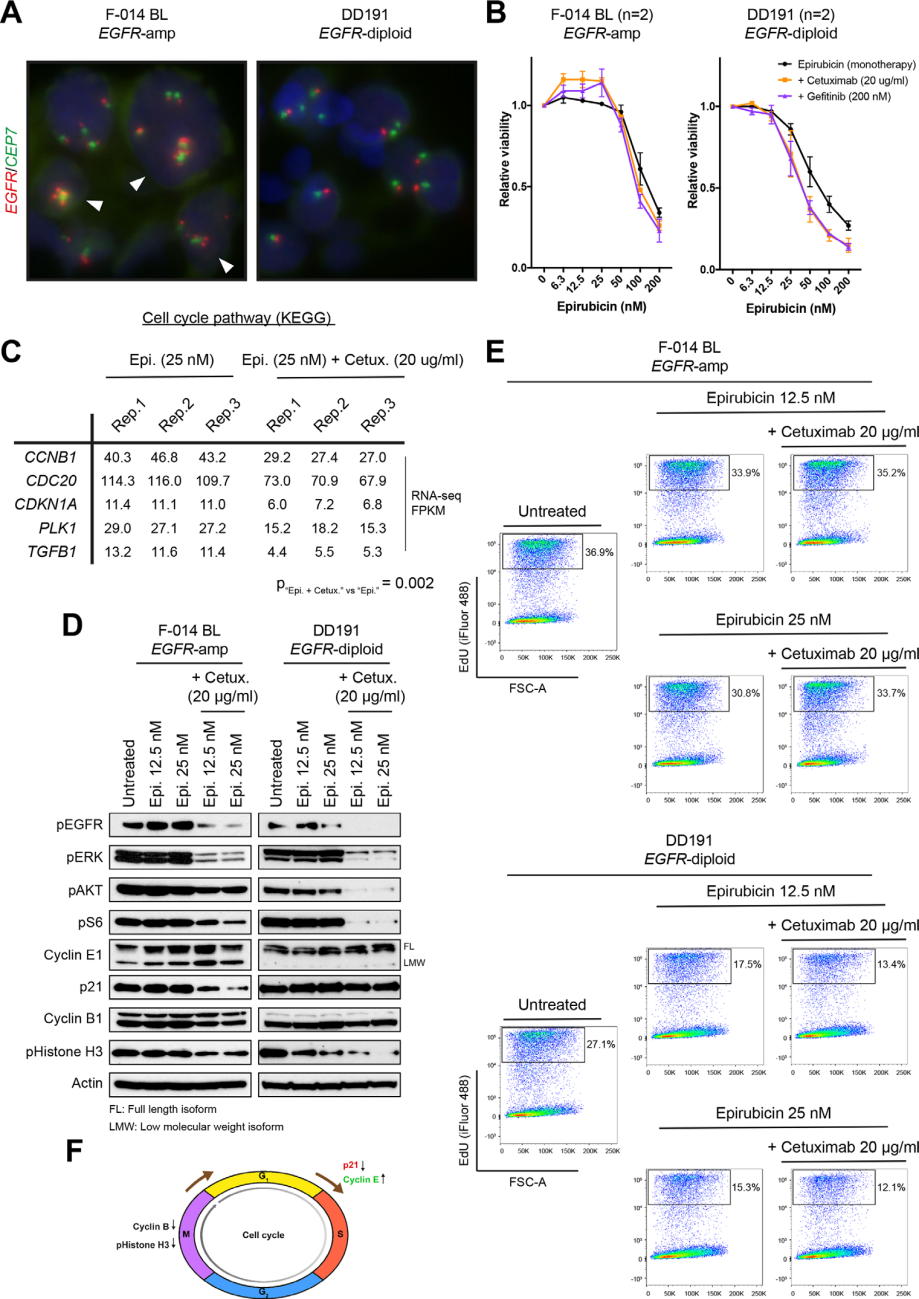
Effect of epirubicin or epirubicin plus EGFR inhibitors in *EGFR*-amplified and non-amplified patient-derived organoids. (A) *EGFR* FISH images, demonstrating gain of copies and diploid status in the F-014 BL and DD191 human GEA PDO lines, respectively. (B) Concentration-dependent effect of epirubicin as a monotherapy or in combination with a stable dose of two different anti-EGFR agents (cetuximab, 20 µg/mL; gefitinib, 200 nM) in the *EGFR*-amplified F-014 BL and *EGFR*-diploid DD191 human GEA PDO lines. The combination of low epirubicin concentrations with anti-EGFR treatments results in a paradoxical increase in viability selectively in the *EGFR*-amplified F-014 BL GEA PDO line. Viability data shown are means±SEM of indicated independent experiments. (C) Pathway analysis of RNAseq data from the *EGFR*-amplified F-014 BL GEA PDO line treated with a low concentration of epirubicin alone or in combination with cetuximab for 24 hours revealed a significant reduction in the expression of cell cycle-related genes associated with the epirubicin and cetuximab combination. RNA from three independent biological replicates were sequenced per condition. (D) Protein analysis of the *EGFR*-amplified F-014 BL and *EGFR*-diploid DD191 human GEA PDO lines treated with two low concentrations of epirubicin alone or in combination with cetuximab for 24 hours. In line with RNAseq data, a reduction in p21 and cyclin B1 protein levels was observed when epirubicin was combined with inhibition of EGFR and downstream MAPK and AKT signalling specifically in the *EGFR*-amplified organoid line. (E) EdU DNA incorporation assay following treatment of the *EGFR*-amplified F-014 BL and *EGFR*-diploid DD191 human GEA PDO lines with two low concentrations of epirubicin alone or in combination with cetuximab for 24 hours. The addition of cetuximab antagonises the antiproliferative effect of epirubicin and accelerates DNA synthesis specifically in the *EGFR*-amplified organoid line. (F) Proposed model of antagonism between epirubicin and anti-EGFR treatments in *EGFR*-amplified GEA. EGFR, epidermal growth factor receptor; FISH, fluorescent in situ hybridisation; FSC-A; forward scatter-area; GEA, gastro-oesophageal adenocarcinoma; KEGG, Kyoto Encyclopedia of Genes and Genomes; PDO, patient-derived organoid.

First, we modelled clinical observations^2 9^ reported in *EGFR*-amplified GEA patients treated with single agent EGFRi (EGFRi), in *EGFR*-amplified PDOs from metastatic gastro-oesophageal cancers (figure 4A) and we showed a significant effect on cell viability on treatment with single-agent anti-EGFR small molecule gefitinib and, to a lesser extent, with the anti-EGFR mAb cetuximab (online supplemental figure 5A).

Next, we combined EGFRi with clinically relevant concentrations of epirubicin^23^ and we observed a consistent increase in cell viability selectively in *EGFR*-amplified PDOs, but not in *EGFR* diploid PDOs (figure 4B and online supplemental figure 5B) or cell lines (online supplemental figure 6). In line with this, pathway analysis of RNA-seq data (online supplemental tables 16 and 17, online supplemental figure 7) from *EGFR*-amplified PDOs treated with epirubicin alone or in combination with cetuximab, showed that the combination of the anthracycline and the anti-EGFR agent significantly downregulated several cell cycle-related genes, including the G1/S negative regulator p21 (encoded by *CDKN1A*) and the M phase-related cyclin B1 (encoded by *CCNB1*; figure 4C). Protein expression analysis of *EGFR*-amplified and non-amplified PDOs treated with epirubicin alone or in combination with cetuximab confirmed that the combinatorial treatment did induce a notable downregulation of both p21 and cyclin B1 proteins selectively in the former, coupled with increased levels of the S phase-promoting cyclin E1 (figure 4D). These data suggested that inhibition of EGFR signalling in an anthracycline treatment background may have a cell cycle-promoting effect in *EGFR*-amplified but not in *EGFR*-non-amplified gastric cancer cells. Indeed, the acceleration of cell cycle on the addition of cetuximab selectively in epirubicin-treated *EGFR*-amplified gastric cancer PDOs was further confirmed using a thymidine analog-based DNA incorporation FACS assay (figure 4E).

Nuclear EGFR has been reported to promote DNA replication and repair, and enhance the expression of various cell cycle-related genes by functioning as a cotranscription factor.^24^ We, therefore, tested whether nuclear EGFR is involved in the increase in proliferation observed in the *EGFR*-amplified PDOs in response to the epirubicin and anti-EGFR combinatorial treatment. Interestingly, cetuximab, both as a single agent and in combination with epirubicin, increased EGFR protein stability, leading to increased EGFR nuclear localisation; however, this cetuximab-mediated increase of nuclear EGFR was observed in both PDOs regardless of their *EGFR* CN status (online supplemental figure 8).

Taken together, our observations suggest that inhibition of EGFR signalling in a background of epirubicin treatment selectively induces the downregulation of the G1/S negative regulator p21 and the upregulation of the S phase-promoting cyclin E1 in *EGFR*-amplified gastric cancer PDOs, thereby leading to increased flux to the S phase of the cell cycle. Cyclin E1 upregulation has been linked to shortened mitosis and increased mitotic exit.^25^ These observations, together with decreased levels of M phase-associated proteins observed when epirubicin is combined with cetuximab (figure 4D), suggest that inhibition of EGFR in a background of epirubicin treatment may also speed up the M-to-G1 transition in *EGFR*-amplified PDOs, thereby leading to an overall accelerated progression through the cell cycle (figure 4F). Increased nuclear localisation of EGFR was observed in cells treated with the combination of epirubicin and cetuximab irrespective of *EGFR* status and, this phenomenon, might partially explain the detrimental effect caused by the addition to panitumumab to EOX in the REAL3 trial population. The activation of additional cotranscriptional factors in *EGFR*-amplified cases might account for the paradoxical effect observed specifically in this subgroup of patients.

## Discussion

In this study, using ddPCR performed on routine diagnostic biopsies obtained from oesophagogastric cancer patients treated in the REAL3 trial, we identify a 7% prevalence of patients with *EGFR*-amplified tumours, which is consistent with the literature.^1–3^ We demonstrate that *EGFR* analysis in plasma is complementary to *EGFR* amplification detection in tumour tissue, and *EGFR* amplification appears associated with poor prognosis, even though these data are not statistically significant likely due to the small number of *EGFR*-amplified cases. Our results also suggest that *EGFR*-amplified tumours did not benefit from anti-EGFR therapy in combination with chemotherapy; in fact, both our clinical and preclinical data suggest an antagonistic effect between anthracyclines and anti-EGFRi, with potential implications for the design of future drug combination trials.

*EGFR* amplification is observed in 6%–10% of gastric and gastro-oesophageal cancers across multiple datasets, and is recognised as negatively prognostic in surgically treated patients.^26–28^ However, the association between *EGFR* amplification and outcome in patients with metastatic gastro-oesophageal cancer treated with cytotoxic chemotherapy in the context of a clinical trial has not been previously described. In the perioperative phase II randomised AIO/CAO STO-0801 trial assessing the addition of panitumumab to perioperative epirubicin, cisplatin and capecitabine chemotherapy in unselected patients with resectable gastro-oesophageal cancer, both EGFR overexpression or gene amplification were negatively prognostic for survival, independent of treatment arm.^21^ Interestingly, the HR for OS in AIO/CAO STO-0801 which used an almost identical chemotherapy regimen (ECX) and the same anti-EGFR therapy as REAL3 (panitumumab) was strikingly similar to the REAL3 results (ECX-P vs ECX HR 1.37, 95% CI 0.84 to 2.25, p=0.2). However, this result lacks statistical power due to the relatively modest size of the AIO/CAO STO-0801 trial (n=160). Notably, the EXPAND trial which investigated the addition of cetuximab to cisplatin and fluoropyrimidine chemotherapy did not use an anthracycline and did not demonstrate a significant detriment to anti-EGFR therapy.^7^ Furthermore, in EXPAND patients with the highest levels of EGFR protein expression appeared to have an increased benefit from cetuximab.^7 29^ Although our data and the retrospective analyses of AIO/CAO STO-0801 and EXPAND trials are based on small numbers of patients with *EGFR*-amplified cancers, they are provocative as, taken together, suggest a negative interaction between anti-EGFR therapy and anthracyclines in *EGFR*-amplified GEA.

Although first generation anti-EGFR trials in unselected GEA patients were negative, hints regarding the potential efficacy of anti-EGFR therapy in biomarker-selected populations have recently emerged in the literature.^2 9 30^ For example, although the COG study of gefitinib compared with best supportive care in unselected previously treated oesophageal cancer patients did not show a benefit in survival for gefitinib, post hoc biomarker analysis using FISH for *EGFR* CN has suggested a significant survival benefit for *EGFR*-amplified tumours (OS benefit for gefitinib in *EGFR*-amplified HR=0.21, 95% CI 0.07 to 0.64; p=0.006).^9 11 30^ Similarly, in a study evaluating multiple EGFRi including ABT-806 and cetuximab in *EGFR*-amplified gastro-oesophageal cancers, several profound responses to single agent anti-EGFR therapy were observed (objective response rate 57%) and the median PFS for patients treated with anti-EGFR therapy alone and in combination was 10 months^2^ and largely surpassed the benefits of standard cytotoxic chemotherapy in gastro-oesophageal cancer.

Heterogeneity of biomarker expression is common in gastro-oesophageal cancer, and can impact significantly on the efficacy of targeted therapy and trial results.^10 31 32^ It is possible that ctDNA sampling may overcome some of the challenges associated with tissue heterogeneity as we have previously shown in colorectal cancer,^33^ however, ctDNA may not be present in all gastro-oesophageal cancer patients as its load relates to tumour burden.^34^ In keeping with this observation, higher ctDNA concentrations were observed in metastatic compared with locally advanced patients in our analysis. Detection of gene amplification in plasma has been suggested to be prognostic for targeting *HER2* and *FGFR2*-amplified gastro-oesophageal tumours^31 35^ and screening for multiple simultaneous amplifications might have important implications. 70% of *EGFR*-amplified patients tested by WGS in our study showed coamplification in other targetable oncogenes such as MET. Future studies using targeted approaches such as nanoString nCounter technology or low-pass WGS might further classify patients into three categories: (1) those whose tumours do not harbour coamplifications and are more likely to benefit from EGFRi; (2) those that carry targetable coamplifications that can be therapeutically exploited in anti-EGFR drug combinations; (3) those with undruggable coamplifications such those in *EGFR* and *KRAS* that are less likely to benefit from EGFRi alone.

The optimal cut-off for sensitivity to anti-EGFR therapy and the value of targeting this axis in *EGFR*-amplified gastro-oesophageal cancer have not yet been fully delineated: Maron *et al* observed responses to anti-EGFR therapy in patients with EGFR CN over the 50th percentile (2.4 copies in plasma).^2^ However, it is plausible that higher levels of *EGFR* amplification (and as such oncogene addiction) could enrich future trials for patients more likely to respond to anti-EGFR therapy.

A potential limitation of this study is that approximately half of REAL3 tumours had tissue available for amplification testing as many samples were used in previous biomarker studies.^36 37^ Specifically, small numbers of *EGFR*-amplified cases were included in the survival analyses limiting the power of this work. That being said, it is worth considering that: (1) the prognostic trend for *EGFR* amplification was also confirmed in liquid biopsies (available for 65% of the ITT cohort) and 200 patients in this analysis did not overlap with those presented for tissue analysis and (2) in multivariate analyses, the effect of *EGFR* amplification on survival appeared to be consistent when adjusted for potential confounders. The second potential limitation of our study is the a priori difference in chemotherapy dose and in survival between the arms of the trial. Patients treated in the EOX-P arm of REAL3 received a dose reduction of oxaliplatin and capecitabine chemotherapy following the results of a safety run-in study. However, we performed our analyses both in the ITT population and by treatment arm in an effort to mitigate this difference. A third consideration relates to the negative interaction between panitumumab and other chemotherapy agents in the REAL3 trial. Indeed, conflicting results are available on the negative interaction between oxaliplatin and cetuximab in metastatic colorectal cancer patients.^38–40^ The observation that a reduction in reactive oxygen species in response to cetuximab treatment can impair oxaliplatin-induced apoptosis provides a biological explanation for these clinical observations.^41^ Whether this hypothesis contributes to explain the inferior outcome observed in EOX-P treated patients in the REAL3 trial remains to be confirmed. However, given durable responses to FOLFOX+ cetuximab have been observed in *EGFR*-amplified metastatic gastro-oesophageal cancer patients^2^ and chemotherapy regimens containing anthracyclines in combination with panitumumab have been consistently associated with inferior outcomes compared with chemotherapy alone,^8 42^ we focused our preclinical analysis on the antagonism between epirubicin and EGFRi.

In conclusion, herein, we present results that suggest that *EGFR* status can be measured using ddPCR in tumour and plasma in a cost-effective way and that *EGFR* amplification is associated with a negative survival outcome in patients with GEA independent of treatment arm. Our results also emphasise the challenge in designing optimal drug combinations in absence of robust patient-centred preclinical models. In view of the relatively rarity of *EGFR*-amplified gastro-oesophageal cancers, we suggest that international collaborative efforts may be required in order to facilitate prospective trials enriched for *EGFR*-amplified tumours based on liquid biopsy testing.

## Data Availability

All data relevant to the study are included in the article or uploaded as online supplemental information. RNA-Seq and WGS data are reported in the online supplemental tables.
