# Supplementary material for: EGFR amplification and outcome in a randomised phase III trial of chemotherapy alone or chemotherapy plus panitumumab for advanced gastro-oesophageal cancers
Source: Gut. 2020 Nov 16;70(9):1632–41. doi: 10.1136/gutjnl-2020-322658 (PMC8355876; doi:10.1136/gutjnl-2020-322658)
Supplement: Supplementary data [file gutjnl-2020-322658supp001.pdf]

SUPPLEMENTARY MATERIALS TO

Title: *EGFR* amplification and outcome in a randomised phase III trial of chemotherapy alone or chemotherapy plus panitumumab for advanced gastroesophageal cancers

Authors: E.C. Smyth, G. Vlachogiannis, S. Hedayat, A. Harwood, S. Hulkki-Wilson, M. Salati, K. Kouvelakis, J. Fernandez-Mateos, G. D. Cresswell, E Fontana, T Seidlitz, C. Peckitt, J.C. Hahne, A. Lampis, R. Begum, D. Watkins, S. Rao, N. Starling, T.S. Waddell, A. Okines, T. Crosby, W. Mansoor, J. Wadsley, G. Middleton, M. Fassan, A. Wotherspoon, C. Braconi, I. Chau, I. Vivanco, A. Sottoriva, D.E. Stange, D. Cunningham & N. Valeri

INDEX

Supplementary Methods 02

Supplementary Figure 1 06

Supplementary Figure 2 07

Supplementary Figure 3 08

Supplementary Figure 4 09

Supplementary Figure 5 10

Supplementary Figure 6 11

Supplementary Figure 7 12

Supplementary Figure 8 13

Supplementary References 14

Supplementary Tables 1-17 provided as excel file

Supplementary Methods

Gastric cancer patient-derived organoids, culture conditions, and drug treatments

The F-014 BL and DD191 gastric cancer PDO lines have been previously described.[12,13] PDO culture and drug treatment assays were conducted as we previously described.[13] Epirubicin and gefitinib were obtained from Selleckhem (Munich, Germany). Cetuximab was obtained from the Royal Marsden Hospital Pharmacy.

Fluorescent in situ hybridisation of formalin-fixed paraffin-embedded samples

Formalin-fixed paraffin-embedded slides were prepared according to a standard protocol of de-paraffinization with xylene, pre-treatment with sodium chloride, digestion with pepsin, and dehydration with ethanol; following this, slides were left to air dry for at least 20 minutes. After a week, the FISH probes [*EGFR* amplification assay: a dual colour assay comprised of a probe mapping to the centromere of chromosome 7 (*CEP7*, spectrum green) and a probe mapping to 7p11.2-p12 (*EGFR*, spectrum orange)] from Vysis (Abbott Molecular, Maidenhead, UK) were applied to the slides for hybridization, according to the manufacturer's instructions; this involved applying the probe sets to the tissue, covering with a glass coverslip, and sealing with rubber cement. The slides were then incubated in a humidified atmosphere at 85 °C for 5 minutes for co-denaturation of probe and target DNA, and subsequently at 37 °C for 16 hours for hybridization. Nuclei were counterstained with 46-diamidino-2-phenylindole (DAPI). Samples were analysed using Zeiss AxioImager Z2 fluorescence microscope, and imaging was done with the Isis fluorescence imaging platform (Metasystems, Cambridge, UK). Tissue sections were scanned for the whole tumour using a marked H&E slide as a guide, and a minimum of 50 nuclei were counted for each sample in a representative area. A primary cut-off of >2 was used to identify EGFR-amplified samples; among these, the mean ratio of *EGFR*:CEN7 was found to be 10 (range: 4-22, median: 8).

Fluorescent in situ hybridization of patient derived organoids

Patient derived organoids were fixed and embedded in paraffin as previously described.[13] Dual-label fluorescent in situ hybridization was performed on organoid sections using the Vysis *EGFR*/*CRP7* probe described above and following standard histological techniques. Briefly, sections were de-paraffinised in Histo-Clear solution (National Diagnostics/Thermo Fisher Scientific, Loughborough, UK) followed by dehydration in absolute alcohol. Organoid sections were then treated with 1% Antigen Retrieval Buffer (TCS Biosciences, Buckingham, UK) for 5 minutes in a boiling pressure cooker, followed by Digest-ALL pepsin (Thermo Fisher Scientific, Loughborough, UK) treatment at 37°C and standard dehydration series prior adding the probe

mix. The probes and target DNA were co-denaturated by incubating at 75ºC for 2 minutes, followed by hybridization at 37ºC overnight. Post-hybridization slides were washed in 0.4x SSC/ 0.3% Igepal at 72ºC for 2 minutes, followed by a wash in 2x SSC/0.1% Igepal at room temperature for 1 minute. Nuclei were counterstained with VECTASHIELD® Antifade Mounting Media (Vector Laboratories, Peterborough, UK) and covered with coverslip. Microscopy and imaging were performed using an Olympus BX61 fluorescence microscope using the SMART Capture imaging software (Digital Scientific, Cambridge, UK).

Digital droplet polymerase chain reaction (ddPCR)

FAM-labelled EGFR (Hs01646307_cn) and VIC-labelled CNTNAP2 (Hs00712117_cn) probes (Thermo Fisher Scientific, Loughborough, UK) were used in a multiplex reaction to assess amplification of *EGFR*. Duplicate PCR reactions were prepared for each sample using the ddPCR supermix for probes without dUTP (Bio-Rad, Watford, UK) and a maximum of 5 ng of template DNA. The PCR reactions were converted into droplets using the QX200 AutoDG Droplet Generator (Bio-Rad, Watford, UK) and the PCR was conducted using the following conditions: 95°C for 10 min; 40 cycles of 94°C for 30 sec and 60°C for 1 min; 98°C for 10 min. The droplets were then analyzed using the QX200 Droplet Reader (Bio-Rad), and the obtained data were analysed using the QuantaSoft software (Bio-Rad). Quality of individual runs was controlled with negative (range 0.6-1.2) and positive (range >20:1) controls, as well as non-template control on each plate. For a valid result, a minimum of 20,000 merged total droplets was required, 400 positive droplets for each assay, and ≤25% difference between replicates.

RNA sequencing and data analysis

RNA sequencing and data analysis were performed by Arraystar (Rockville, MD, USA). Library construction, sequence quality control, and data analysis were performed as previously described.[13]

EdU incorporation assay

F-014 BL and DD191 organoids were harvested and dissociated following the passaging procedure previously described.[13] Single cells were seeded in wells of 24-well plate (50,000 cells per well, embedded in 120 ul of matrigel) and overlayed with 1 ml of culture media. Two days post seeding the overlaying media was removed and replaced with 1 ml of drug containing media. 24h post treatment, EdU (Abcam, Cambridge, UK) was added in the culture media at a final concentration of 20 uM. Plates were placed back in the incubator and organoids were incubated in the presence of EdU for another 4h before being harvested and converted into single cells. Resulting cell suspensions were pelleted, washed with a 3% BSA solution in PBS/EDTA 1mM, passed through a 70 µm cell strainer (Sigma- Aldrich, Gillingham,

Smyth EC, *et al. Gut* 2021; 70:1632–1641. doi: 10.1136/gutjnl-2020-322658

UK) in order to eliminate cell clumps, and fixed according to the manufacturer's instructions. Further washes, cell permeabilization, and staining with iFluor488 were performed according to the manufacturer's instructions. Stained cells were analysed using the BD LSR II flow cytometer (BD Biosciences, San Jose, CA, USA), and the data generated were analysed with the FlowJo software.

Western blot

Protein extraction and western blotting was performed as we previously described.[13] The antibodies used in the present manuscript are listed in the table below:

Target	Supplier	Cat. No.	Dilution
Actin	Cell Signaling	3700	1:5000
pAKT (Ser473)	Cell Signaling	4060	1:1000
Cyclin B1	Cell Signaling	12231	1:1000
Cyclin E1	Cell Signaling	20808	1:1000
pEGFR (Tyr1068)	Cell Signaling	3777	1:1000
pERK1/2 (Thr202/Tyr204)	Cell Signaling	4370	1:1000
pHistone H3 (Ser10)	Cell Signaling	3377	1:1000
p21	Cell Signaling	2947	1:1000
pS6 (Ser235/236)	Cell Signaling	4858	1:1000

Low-pass whole-genome sequencing and data analysis

For FFPE-extracted material, 20 ng of DNA were initially repaired for downstream library preparation following the NEBNext® FFPE DNA Repair Mix protocol (New England Biolabs); WGS libraries were then constructed using the NEBNext® Ultra II^{TM} FS DNA Library Prep Kit for Illumina (New England Biolabs), starting with an enzymatic fragmentation for 10 minutes. For plasma-extracted material, WGS libraries were constructed directly from 5 ng of cfDNA using the NEBNext® Ultra^{TM} DNA Library Prep Kit for Illumina (New England Biolabs). All libraries were indexed with unique indexing primers using the NEBNext® Multiplex Oligos for Illumina® Dual Index Primers Set I (New England Biolabs). Quality of constructed libraries was assessed using TapeStation (Agilent Genomics), and library quantification was done with the Qubit® Fluorometer (Life Technologies). Pooled libraries were sequenced (50 bases paired-end) on an Illumina NovaSeq 6000 system (Illumina).

Smyth EC, *et al. Gut* 2021; 70:1632–1641. doi: 10.1136/gutjnl-2020-322658

FASTQ files were trimmed using skewer[43] for adaptor content with a minimum length allowed after trimming of 35 bp, only on reads with a minimum mean quality of 10 and with the filter to remove highly degenerative reads (-l 35 -Q 10 -n). Trimmed FASTQ files were aligned using bwa mem[44] to hg38 (GRCh38). Sam files were sorted, compressed to bam files and duplicates were marked using Picard tools (www.broadinstitute.github.io/picard/). Indexing was performed using samtools.[45] Bam files were then processed using QDNAseq[46] to convert read counts in 500kb bins across the autosomes of hg38 into log2ratio data. Data normalisation was performed in accordance to the QDNAseq workflow, except for the step which uses the *smoothOutlierBins* function which was seen to artificially depress signal from highly amplified bins. Bins for hg38 were also generated according to QDNAseq instructions. Bins with a log2ratio greater than or equal to 0.58 were considered amplified. Genes present within these bins were identified using biomaRt.[47]

Supplementary Figure 1

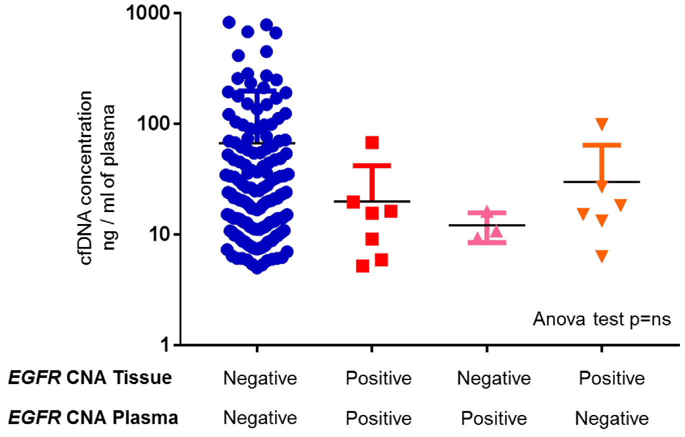

Supplementary Figure 1. cfDNA concentration according to *EGFR* copy number alterations (CNA). No significant differences in cfDNA concentrations were observed among patients with or without EGFR amplification in either plasma cfDNA or tissue.

cfDNA= cell-free DNA

Supplementary Figure 2

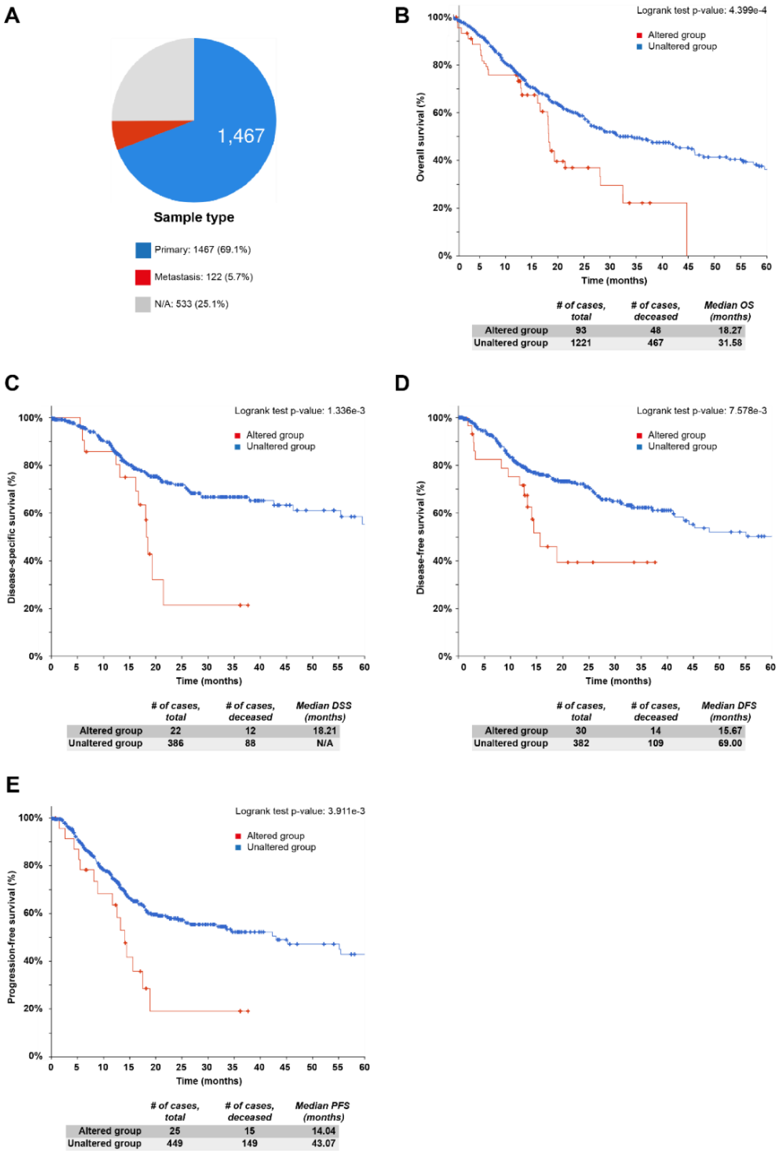

Supplementary Figure 2. *EGFR* status and clinical outcome in independent patient cohorts. The association between *EGFR* amplification and clinical outcome was tested in 2122 samples from 2054 patients with localised or metastatic GEA (**A**) using the cBioportal for Cancer Genomics website (https://www.cbioportal.org). Presence of *EGFR* amplification (altered group in red) correlated with Overall Survival (**B**), Disease-Specific Survival (**C**), Disease-Free (**D**) and Progression-Free Survival (**E**).

Supplementary Figure 3

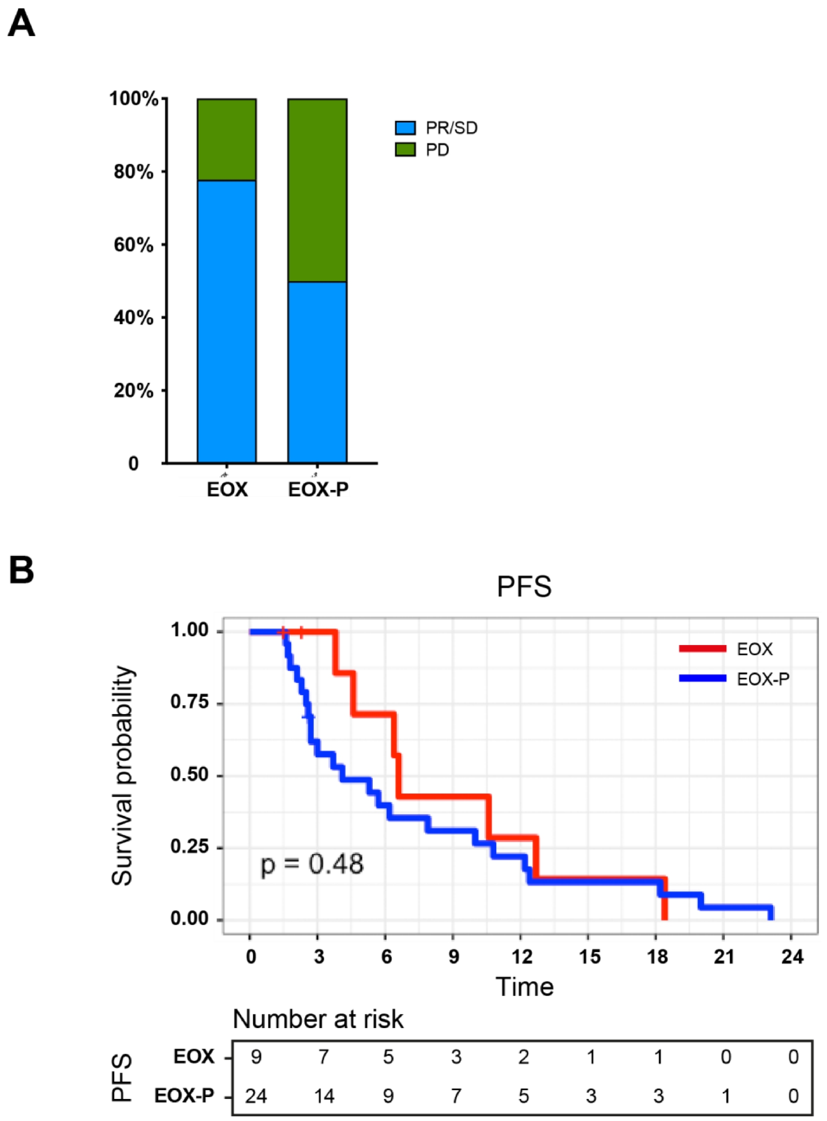

Supplementary Figure 3. Response to treatment in *EGFR*-amplified patients. Overall response rate (**A**) and progression-free survival (PFS) (**B**) in all *EGFR*-amplified patients based on detection of EGFR amplification detected in tissues and/or plasma cell-free DNA.

EGFR=epidermal growth factor receptor; PR=partial response; SD=Stable Disease; EOX= epirubicin + cisplatin + capecitabine; P= panitumumab.

Supplementary Figure 4

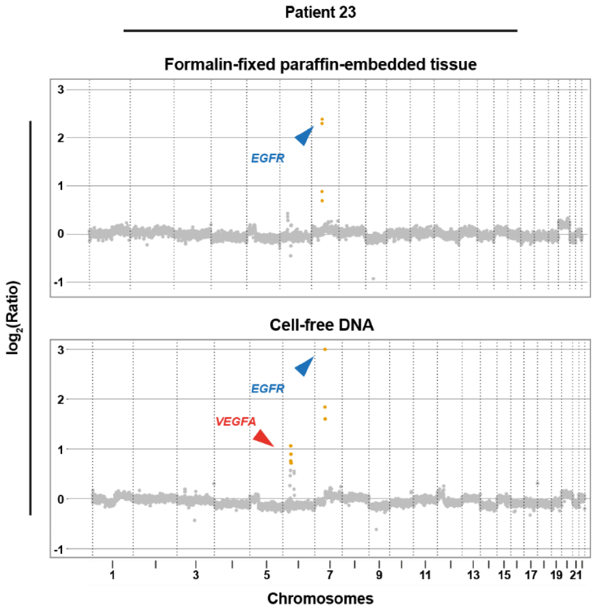

Supplementary Figure 4. Comparison of low-pass whole genome sequencing (WGS) in tissue and plasma from the same patient. WGS documented the *EGFR* amplification in both tissue and circulating cell-free DNA (cfDNA) in a patient treated with the combination of chemotherapy and the EGFR inhibitor panitumumab. WGS analysis of cfDNA also revealed a *VEGFA* amplification likely associated with resistance to EGFR inhibitors that was not detected in the matching tissue.

EGFR=epidermal growth factor receptor; VEGFA=Vascular endothelial growth factor A

Smyth EC, *et al. Gut* 2021; 70:1632–1641. doi: 10.1136/gutjnl-2020-322658

Supplementary Figure 5

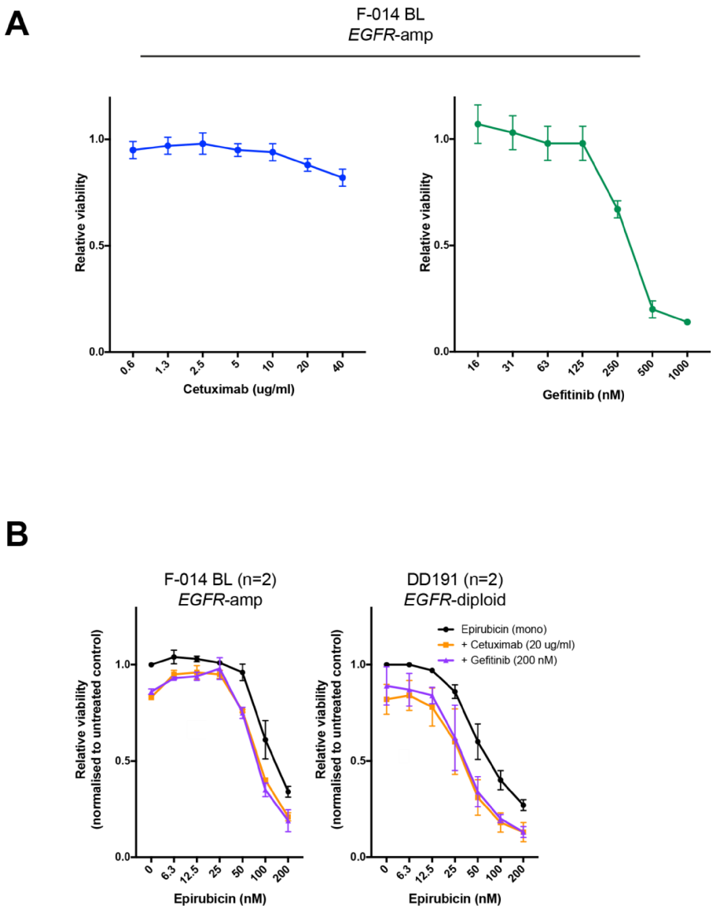

Supplementary Figure 5. Response to EGFR inhibitors alone or in combination with chemotherapy in patient-derived organoids (PDOs). (**A**) *EGFR*-amplified PDOs (F014-BL) were treated with single agent cetuximab or gefitininb. (**B**) *EGFR*-amplified (F014-BL) and non-amplified (DD191) PDOs were treated with increasing concentrations of epirubicin alone or epirubicin in combination with anti-EGFR agents. Both cetuximab and gefitinib show similar inhibition of cell viability.

EGFR=epidermal growth factor receptor

Supplementary Figure 6

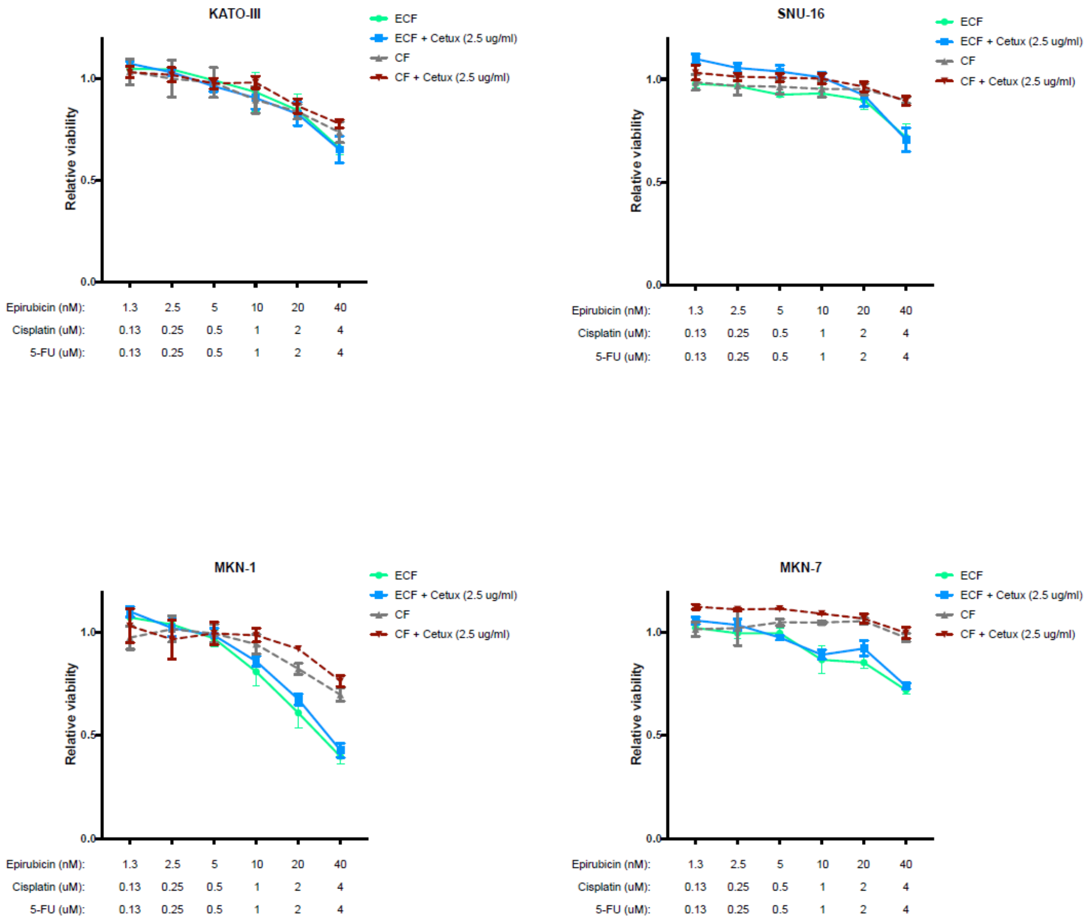

Supplementary Figure 6. Effect of chemotherapy doublet or triplet with or without cetuximab on cell viability in *EGFR* diploid cell lines. Cells were treated for 72 hours with increasing concentrations of Epirubicin (E), Cisplatin (C) and 5-Fluorouracil (F). In MKN-1 and MKN-7 cells, addition of Epitubicin to Cisplatin and 5-FU significantly decreased cell viability. In none of the cell lines the addition of cetuximab to either doublet or triplet chemotherapy caused synergic or antagonistic effect on cell viability compared to chemotherapy alone.

Smyth EC, *et al. Gut* 2021; 70:1632–1641. doi: 10.1136/gutjnl-2020-322658

Supplementary Figure 7

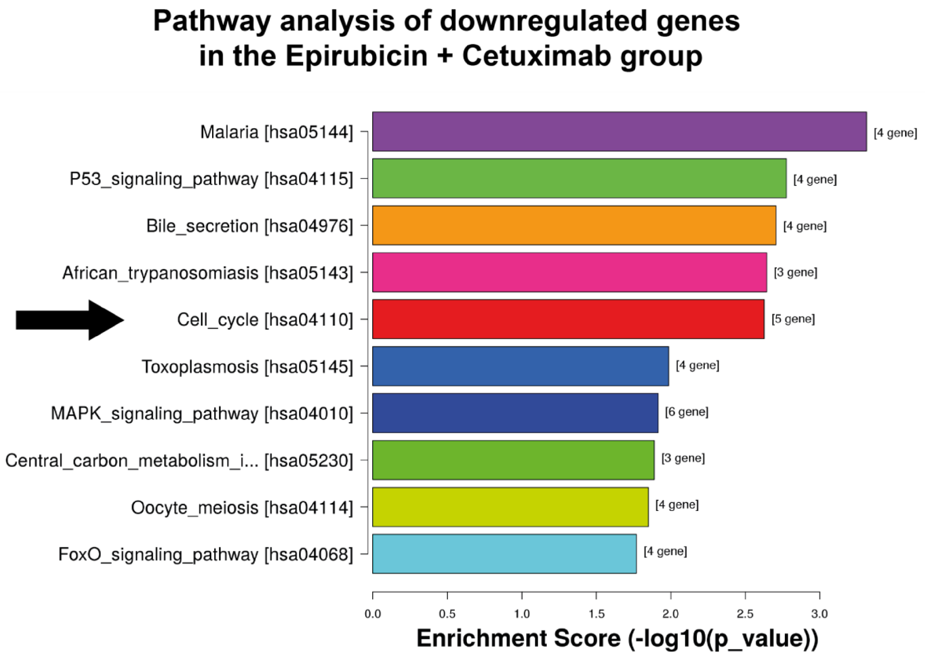

Supplementary Figure 7. Pathway analysis in *EGFR*-amplified organoids treated with epirubicin plus cetuximab versus epirubicin alone. Horizontal bars show pathway and number of genes enriched in organoids treated with the combination of anthracycline and anti-EGFR.

EGFR=epidermal growth factor receptor

Supplementary Figure 8

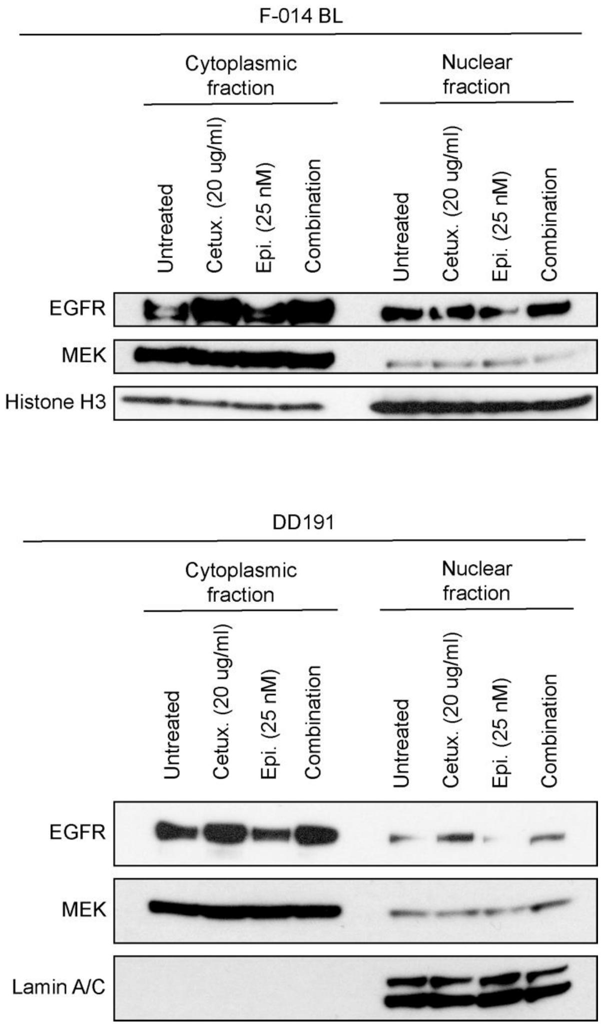

Supplementary Figure 8. Sub-cellular localization of EGFR in GEA PDOs following treatment with epirubicin, cetuximab, and their combination. Cetuximab, both as a single agent and in combination with epirubicin, stabilizes EGFR in human GEA PDOs regardless of their EGFR copy number status, thereby inducing increased nuclear localization of EGFR.

Supplementary References

43. Jiang H, Lei R, Ding S-W, *et al.* Skewer: a fast and accurate adapter trimmer for next-generation sequencing paired-end reads. BMC Bioinformatics. 2014;15:182. doi: 10.1186/1471-2105-15-182.
44. Li H, Durbin R. Fast and accurate short read alignment with Burrows-Wheeler transform. Bioinformatics. 2009;25:1754–1760. doi: 10.1093/bioinformatics/btp324.
45. Li H, Handsaker B, Wysoker A, *et al.* The sequence Alignment/Map format and SAMtools. Bioinformatics. 2009;25:2078–2079. doi: 10.1093/bioinformatics/btp352.
46. Scheinin I, Sie D, Bengtsson H, *et al.* DNA copy number analysis of fresh and formalin-fixed specimens by shallow whole-genome sequencing with identification and exclusion of problematic regions in the genome assembly. Genome Res. 2014;24(12):2022-2032. doi:10.1101/gr.175141.114
47. Durinck S, Spellman PT, Birney E, *et al.* Mapping identifiers for the integration of genomic datasets with the R/Bioconductor package biomaRt. Nat Protoc. 2009;4(8):1184-1191. doi:10.1038/nprot.2009.97

Smyth EC, *et al. Gut* 2021; 70:1632–1641. doi: 10.1136/gutjnl-2020-322658
